# Hijacking the Supplies: Metabolism as a Novel Facet of Virus-Host Interaction

**DOI:** 10.3389/fimmu.2019.01533

**Published:** 2019-07-03

**Authors:** Katharina A. Mayer, Johannes Stöckl, Gerhard J. Zlabinger, Guido A. Gualdoni

**Affiliations:** ^1^Institute of Immunology, Center of Pathophysiology, Infectiology and Immunology, Medical University of Vienna, Vienna, Austria; ^2^Division of Nephrology and Dialysis, Department of Medicine III, Medical University of Vienna, Vienna, Austria

**Keywords:** virus, metabolism, rhinovirus, host-pathogen interaction, metabolome

## Abstract

Viral replication is a process that involves an extremely high turnover of cellular molecules. Since viruses depend on the host cell to obtain the macromolecules needed for their proper replication, they have evolved numerous strategies to shape cellular metabolism and the biosynthesis machinery of the host according to their specific needs. Technologies for the rigorous analysis of metabolic alterations in cells have recently become widely available and have greatly expanded our knowledge of these crucial host–pathogen interactions. We have learned that most viruses enhance specific anabolic pathways and are highly dependent on these alterations. Since uninfected cells are far more plastic in their metabolism, targeting of the virus-induced metabolic alterations is a promising strategy for specific antiviral therapy and has gained great interest recently. In this review, we summarize the current advances in our understanding of metabolic adaptations during viral infections, with a particular focus on the utilization of this information for therapeutic application.

## Cellular Metabolism: the Novel Frontier of Host–Pathogen Interaction

Viruses depend on the host cell to obtain the macromolecules and biosynthesis machinery required for their replication. In order to ensure the undisturbed supply of these elements, viruses have evolved a plethora of strategies to shape host-cell metabolism according to their specific needs. The simultaneous course of both the activation of host cell defense mechanisms and the high biomolecular turnover associated with virion production results in a highly anabolic cellular state. This is often accompanied by upregulation of the ingestion of an extracellular carbon source (e.g., glucose or glutamine) and a redirection of these carbon supplies to metabolic pathways crucial for viral replication, such as lipogenesis and nucleotide synthesis. However, not only do viruses shape host-cell metabolism in order to obtain supplies for virion production, but they also induce a reorganization of the cellular membrane and biosynthesis machinery, which is accompanied by alterations in lipid metabolism, as we shall explain later.

The first insights into the dependence of viruses on certain carbon sources were gained decades ago, when researchers focused on investigating the consequences of glucose or glutamine deprivation on viral replication (1–6). However, it was the availability of mass spectrometry (MS)-based analysis of the metabolome that enabled fast progress toward an in-depth understanding of the interaction between viruses and host-cell metabolism. Munger et al. pioneered the field in 2006, when they showed that human cytomegalovirus (HCMV) not only was highly dependent on extracellular carbon but also induced a plethora of alterations in host-cell metabolism that are required for proper replication (7). MS-based assessment of the host cell metabolome and carbon flux has since then become widely available and has enabled the investigation of host–pathogen interactions in detail. Furthermore, the acquired knowledge on these processes has enabled the establishment of several antiviral strategies, and the exploitation of the novel metabolic insights in terms of therapy has only just begun. Herein, we review the recent progress made toward our understanding of the interactions between viruses and host-cell metabolism, and we will also elaborate on strategies that might result in targeted antiviral therapy.

## Glucose and Glutamine: Virus-Induced Feeding of the Tricarboxylic Acid Cycle

Under homeostatic and aerobic conditions, cells maintain their energy production mainly by aerobic glycolysis, which is followed by feeding pyruvate into the tricarboxylic acid (TCA) cycle and subsequent utilization of reduced molecules in oxidative phosphorylation. However, under anaerobic conditions, pyruvate is converted to lactate, which is then eliminated by efflux from the cell. Aside from anaerobic conditions, this phenomenon can often be observed even under normal oxygen conditions, as was first described in cancer cells by Otto Warburg and has thus been termed the Warburg effect (8). Under these circumstances, the intermediates of the TCA cycle are mainly fed into anabolic processes, such as lipogenesis. Cells infected by certain viruses appear to adopt similar metabolic alterations in order to cope with the high anabolic demands of virion production. Nonetheless, there are highly unique patterns of virus-induced reshaping of host cell metabolic processes and the mode of manipulation appears to be different between DNA and RNA viruses.

### DNA Viruses

Members of the *Herpesviridae* are probably the best-studied group of viruses in terms of their impact on cellular metabolism. Herpes simplex virus-1 (HSV-1) was among the first viruses for which a dependency on extracellular glucose was shown. Deprivation of glucose from the medium had detrimental effects on virion production, whereas glutamine appeared to be more dispensable for the replication of this virus (2). Later studies confirmed the dependency of HSV-1 on glucose, as the glycolysis inhibitor 2-deoxyglucose (2-DG) also impaired viral replication (4, 9). 2-DG is a glucose analog that impairs the function of phosphoglucose isomerase and thus results in both an inhibition of glycolysis and the processing of glucose toward the TCA cycle (in contrast to the more downstream inhibitor oxamate that inhibits only anaerobic glycolysis). Abrantes et al. found that HSV-1 increased the glucose uptake, lactate efflux, and ATP content of HSV-1 infected cells, which was accompanied by an activation and enhanced expression of phosphofructokinase-1, a rate-limiting enzyme in glycolysis (10). In contrast, using a metabolomic screening, Vastag et al. found that glycolysis was not markedly induced by HSV-1, and the virus instead triggered anaplerotic (glutamine-dependent) feeding of the TCA cycle and an enhancement of pyrimidine synthesis (11). A possible explanation could be that the uptake of glucose mainly shifted to nucleotide synthesis pathways rather than glycolysis or the TCA cycle, which was further underlined by the increase in pentose phosphate pathway intermediates in the metabolomic analysis and would explain the high susceptibility of this virus to nucleotide analog treatment (11).

HCMV, another important member of the *Herpesviridae* family, causes significant morbidity in immunosuppressed individuals (12–14). Since the metabolic alterations caused by this virus have already been extensively reviewed recently (15), we will only briefly discuss the main findings in order to better delineate the concepts of differential metabolic alterations by viruses. Early investigations had hinted toward a manipulation of host-cell metabolism by HCMV, where it was shown that glucose uptake was enhanced in infected cells (5). In the first metabolomic study of virus-infected cells conducted by Munger et al. the authors were able to show that metabolites from glycolysis, TCA cycle, and pyrimidine pathways were increased upon infection, which was accompanied by the upregulation of enzymes involved in these pathways (7). Further carbon flux analysis delineated how an increase in glucose uptake results in a fast processing through glycolysis and the TCA cycle toward fatty acid (FA) biosynthesis (16). The expansion of pyrimidine metabolite pools was found to be of particular importance for the correct glycosylation of viral proteins, as pyrimidine feeds into glycosylation pathways via UDP-sugars (17). Mechanistically, early HCMV gene expression was shown to be responsible for the changes in glycolytic flux and appeared to be dependent on Ca^++^ signaling, since calmodulin-dependent kinase kinase (CaMKK) inhibition abolished the HCMV-induced metabolic alterations (9). Subsequent research has highlighted a role of AMP-activated protein kinase (AMPK) in the replication cycle of HCMV, since this kinase is activated upon infection and its inhibition has detrimental effects on viral replication (18, 19). Since CaMKK is known to be upstream of AMPK, and blocking of CaMKK abolished the HCMV-induced AMPK activation, the authors proposed a CaMKK–AMPK axis in the mediation of HCMV's metabolic effects. Other groups have investigated the role of glucose transporters (GLUTs) in HCMV infection and found upregulation of GLUT4 expression, but downregulation of GLUT1, following infection (20). These changes in GLUT expression were later shown to be dependent on the carbohydrate-response element-binding protein (ChREBP), which is targeted in HCMV infection (21). Apart from the apparent need for adequate glucose supply, HCMV also depends on extracellular glutamine as a carbon source (22). Deprivation of glutamine from the extracellular medium dampened high-titer virus replication, which could be restored by the addition of TCA cycle metabolites, thus pointing toward an anaplerotic utilization of glutamine in HCMV infection (22). Recent research has additionally established a role of the viral protein U_L_38 in the upregulation of both glucose and glutamine (and other amino acid) consumption, which was mediated by the modulation of tuberous sclerosis complex 2 (TSC2) but was mTOR independent (23).

Epstein-Barr virus (EBV) causes infectious mononucleosis, and its latent infection is associated with the development of various malignant diseases. Latently infected cells were found to enhance both glucose and glutamine uptake and to have deregulated glycolysis (24, 25). These changes were described to have been induced by EBV's latent membrane protein 1 (LMP1) and were associated with fibroblast growth factor receptor 1 (FGFR1) signaling (24, 25). Such metabolic alterations have been speculated to play a role in the long-term cancerogenic transformation of the latently infected cells (25).

Another virus of the *Herpesviridae* family, Kaposi's sarcoma-associated herpesvirus (KSHV), was also shown to broadly interact with host-cell metabolism in a quite sophisticated manner. Sanchez et al. found that glucose and glutamine were important for early viral replication and gene translation, respectively (26, 27). Furthermore, FA synthesis was shown to be crucial for optimal virus assembly and maturation (27, 28). Yogev et al. found that viral-encoded microRNAs were important for inducing the alterations in glucose metabolism, by repressing the expression of the metabolic regulator genes *EGLN2* (encoding Egl nine homolog 2) and *HSPA9* (encoding Stress-70 protein, mitochondrial), which then results in increased glycolysis and GLUT1 expression (29). Additionally, recent evidence suggests that KSHV-transformed cells critically depend on extracellular glutamine and asparagine to enable ɤ-nitrogen synthesis that fuels nucleotide synthesis (30). Accordingly, expression of enzymes engaged in glutamine metabolism including glutaminase, glutamate dehydrogenase 1, and glutamic-oxaloacetic transaminase 2 were needed to support cell proliferation in KSHV-transformed cancer cells (30). Supporting this evidence, the research group of Chandran was able to demonstrate that both *de-novo* and latent KSHV infection of endothelial cells and B cells induces glutaminase expression, which was found to be partly c-Myc dependent. Furthermore, the virus triggers extracellular glutamate secretion, the breakdown product of glutaminase-mediated enzymatic degradation of glutamine (31). The authors proposed that glutamate may act as an autocrine and paracrine growth factor during the course of KSHV-induced oncogenic transformation, as blockade of glutamate secretion or inhibition of metabotropic glutamate receptors attenuated KSHV-infected cell proliferation (31). Other important targets within the host cell that shape the KSHV anti-viral response and/or KSHV-induced cell proliferation include HECT domain and ankyrin repeat containing E3 ubiquitin protein ligase 1 (HACE1) (32) and heme oxygenase-1 (33).

The group of Christofk has performed pioneering work toward our better understanding of adenovirus-induced host cell reprogramming and particularly in the mechanistic basis of virus–metabolome interactions. They were able to show that the viral product E4ORF1 localizes to the nucleus and binds the transcription factor Myc to induce the transcription of a number of glycolytic genes, resulting in enhanced glycolytic pathway activity and nucleotide production (34). Later, they showed how Myc regulated glutamine metabolism in adenovirus-infected cells and that glutaminase was a critical enzyme for adenovirus replication, which was also true for HSV-1 and influenza A (35). Coherently, inhibition of glutaminase by CB-839 impaired adenovirus, HSV-1, and influenza A replication (35).

**Figure 1:**
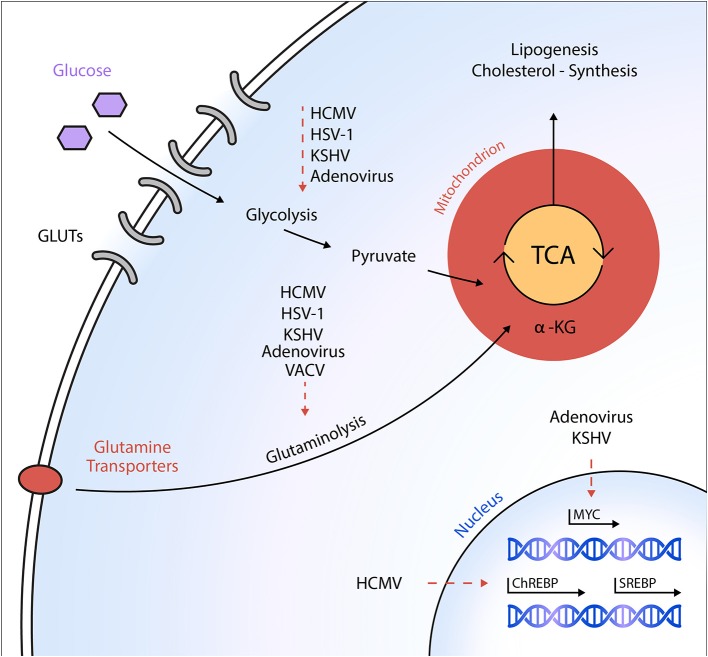
Schematic overview of metabolic targets of DNA viruses. Different DNA viruses activate specific anabolic metabolic programs in host cells to ultimately support viral replication and virion maturation. Dashed arrows indicate a virus-mediated activation of the respective metabolic pathway or an activation of the transcription factor, respectively. HCMV, human cytomegalovirus; HSV-1, herpes simplex virus-1; KSHV, Kaposi's sarcoma-associated herpesvirus; VACV, vaccinia virus; GLUT, glucose transporter; ChREBP, carbohydrate-response element-binding protein; SREBP, sterol regulatory element-binding protein; α-KG, α-ketoglutarate; TCA, tricarboxylic acid cycle.

All DNA viruses discussed so far induce glycolysis and/or increase glucose uptake in the course of infection. However, an exception to this is the vaccinia virus (VACV). Metabolomic studies have shown that although the virus does not affect glycolytic flux, it is highly dependent on glutamine as a carbon source for feeding into the TCA cycle (36, 37) (Figure 1). Further studies showed that the viral protein C16 might be responsible for these effects through the stabilization of hypoxia-inducible factor 1-alpha (HIF-1α) (38).

### RNA Viruses

**Figure 2:**
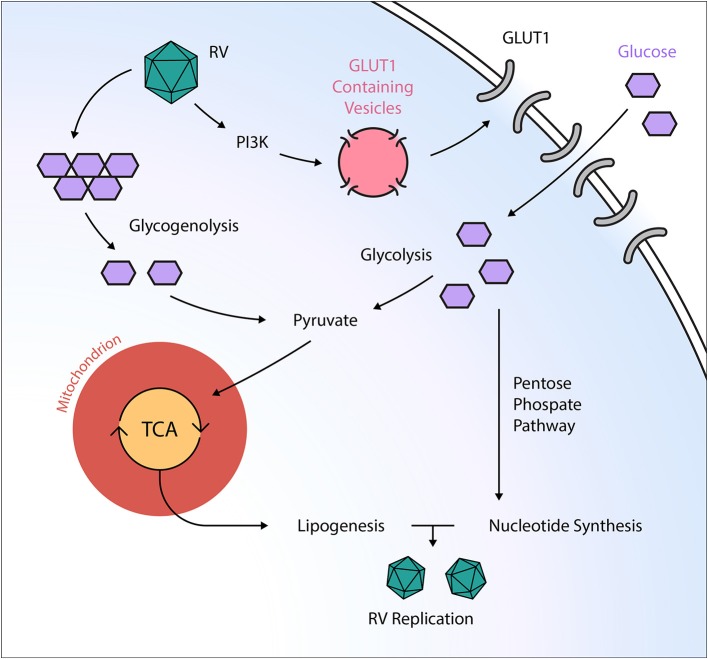
Post-transcriptional manipulation of the host cell metabolism by the RNA virus rhinovirus. Rhinovirus (RV) orchestrates an anabolic reprogramming of the host cell metabolism: RV induces PI3K-dependent trafficking of GLUT1-containing vesicles to the cell membrane, ultimately leading to increased glucose uptake. Subsequently, RV increases both glycolysis and glycogenolysis, providing TCA intermediates needed for anabolic lipogenesis. Additionally, RV infection activates the pentose phosphate pathway, resulting in elevated nucleotide levels that support viral replication. GLUT, glucose transporter; PI3K, phosphatidylinositol 3-kinase; RV, rhinovirus; TCA, tricarboxylic acid cycle.

In contrast to the large DNA viruses discussed above, we found a markedly different mode of metabolism manipulation by the small RNA virus rhinovirus (RV), which belongs to the *Picornaviridae* family and is the causative agent of the common cold. Similar to other viruses, we found an enhancement of glucose uptake and the virus was dependent on both extracellular glucose and glutamine for optimal viral replication (39). However, the amplification of glucose uptake was detectable as fast as 1.5 h upon infection, which ruled out a transcriptional control of this process. Indeed, we found the enhanced uptake to be reversible by phosphoinositide 3-kinase (PI3K) inhibition, suggesting a role of this pathway in mediating RV's effects. In contrast to HCMV infection, we found upregulation of GLUT1 expression upon RV infection, whereas GLUT3 expression was unaffected (Figure 2). This is in line with the concept of PI3K-driven upregulation of GLUT1, likely to mediate RV effects. Metabolomic studies revealed increased levels of metabolites associated with glycogenolysis, a process that has not been described so far in the context of viral infections. Furthermore, we found an enhancement of lipogenesis and nucleotide synthesis. The deprivation of both glutamine and glucose from the medium impaired high-titer RV replication, and the early glycolysis inhibitor 2-DG potently inhibited viral replication and reversed the RV-induced alterations of the host cell metabolome. Thus, our findings underline the potential of metabolism as a target of antiviral therapy (39).

As stated above, glutaminase is of pivotal importance for influenza virus replication (35). Furthermore, the influenza virus was shown to depend on extracellular glucose, and viral replication could be impaired by treatment with the glycolysis inhibitor 2-DG (6). In contrast, recent work has found that 2-DG has detrimental effects on survival in an *in vivo* influenza infection, which was attributed to an unregulated unfolded protein response in the absence of glucose (40). These findings are particularly intriguing, as they show how a metabolism-targeting intervention that is effective on the cellular level might still be detrimental when applied systemically and could thus affect a plethora of different cell populations. These considerations need to be taken into account when designing metabolism-targeting antivirals and deciding on their route of administration.

Several viruses of the *Flaviviridae* family were shown to be potent modulators of host-cell metabolism. Zika virus was shown to modulate metabolism differently in human and mosquito cells; that is, whereas the infection resulted in enhanced glucose utilization through the TCA cycle in human cells, glucose utilization shifted toward the pentose phosphate pathway in mosquito cells (41). These differences resulted in a reduction of nucleotide triphosphates and AMPK-dependent cell death in human cells (41). Dengue virus (DENV) stimulates and requires glycolysis for optimal replication (42), which was found to be mediated by the induction of glyceraldehyde-3-phosphate dehydrogenase (GAPDH) by the virus's non-structural protein NS1 (43). The distantly related hepatitis C virus (HCV) was also shown to increase glucose demand and enhance glycolysis in infected cells (44, 45). Interestingly, HCV appears to have evolved several strategies to target host cell glycolysis, where it was shown that the NS5A protein interacts with hexokinase 2 to increase the glycolytic flux (45), and the HCV-regulated microRNA 130a enhances the activity of pyruvate kinase, another key enzyme in glycolysis (46).

Recently, there has also been great progress in the elucidation of the metabolic requirements for human immunodeficiency virus (HIV) replication. Hollenbaugh et al. were among the first to study the metabolic alterations in HIV-1-infected cells by means of metabolomics (47). Intriguingly, they were able to show that HIV-1 induced marked changes depending on the infected cell type; that is, whereas CD4^+^ T cells exhibited increased glucose uptake and metabolite pools in the TCA cycle, the opposite was found for infected macrophages (47). Subsequent investigations have confirmed the increase in glucose uptake by infected CD4^+^ T cells (48–53). These alterations were shown to be accompanied by increases in the expression of the glucose transporters GLUT1 (51–53), GLUT3 (53), GLUT4 (53), and GLUT6 (53), and also by increases in the expression of the key glucose-processing enzyme hexokinase 1 (HK1) (53, 54). Furthermore, phospholipase D1 (PLD1) was found to be a crucial regulator of the HIV-1-induced metabolic alterations in CD4^+^ T cells (48). PLD1 further induced the activation of c-Myc, resulting in the activation of a transcriptional program that led to enhanced glucose uptake and nucleotide biosynthesis. Consequently, the pharmacologic inhibition of PLD-1 led to a reduction in HIV-1 replication (48). Apart from glucose, glutamine concentrations were found to be elevated as well in HIV-1-infected CD4^+^ T cells, which was accompanied by increases in the levels of glutaminase (55). Studying the differences between the alterations induced by HIV-1 and HIV-2, Hollenbaugh et al. found that although both viruses induced similar changes in infected macrophages, there were differences observed in the levels of quinolinate, a tryptophan pathway component (56). In another important study, Hegedus et al. found marked differences between primary T cells and cell lines infected with HIV-1, thus underlining the importance of the cell system when studying cellular metabolomics (49).

Taking these findings together, we can see that most viruses have evolved strategies to alter central carbon supply pathways, such as glucose or glutamine consumption, and these manipulations were shown to be vital for high-titer virus replication. Apart from this, virion production requires a re-orchestration of the entire biosynthesis machinery, a process that usually involves a reorganization of many parts of the cellular lipidome, as we review in the next section.

## Viral Control of Fatty Acid Metabolism

Apart from the alterations mentioned above, the FA synthesis machinery of the host cell has proven vital for viral genome replication, virion production and morphogenesis. Several viruses induce the formation of phosphatidylinositol 4-phosphate/cholesterol-enriched membranes to build viral replication complexes (VRCs) at the interface of the host endoplasmic reticulum (ER). Accumulation of sterols at the VRCs of RNA viruses allows for the production of secluded membranes that contain an optimal environment for viral replication and shield virus nucleic acids from immune surveillance (i.e., cytosolic pattern recognition receptors) (57, 58). Formation of the VRCs critically depends on reprogramming of the host's sterol synthesis via recruitment of the phosphatidylinositol-4 kinase III beta and oxysterol-binding protein (PI4KB–OSBP) axis, and disruption of cellular cholesterol homeostasis impairs viral replication (59–63). Apart from this apparent need for an adequate sterol supply, viruses transactivate and co-opt enzymes engaged in *de novo* lipid synthesis and in the enzymatic modification of intracellular FAs.

Generally, several carbon sources can be used as substrates for FA or cholesterol synthesis, with the most important one being citrate derived from the TCA cycle. Citrate is carried across the mitochondrial membrane and cleaved into acetyl-CoA in the cytosol. Acetyl-CoA is then carboxylated by acetyl-CoA carboxylase (ACC) to yield malonyl-CoA. FA synthase (FASN) catalyzes the production of palmitic acid (C16:0) from cytosolic acetyl-CoA and malonyl-CoA in the presence of NADPH. The palmitic acid can then be further processed by elongases and desaturases into more complex FAs for use in the synthesis of cell membranes, storage in lipid droplets, or the palmitoylation of host and viral proteins. For sterol biosynthesis, two units of acetyl-CoA are metabolized to form acetoacetyl-CoA, which then enters the mevalonate pathway. On the other hand, FAs can be metabolized by catabolic beta-oxidation to yield high amounts of ATP.

Several key metabolic transcription factors activate the transcriptional program of anabolic lipid and sterol metabolism. Among those, sterol regulatory element-binding proteins (SREBPs) represent the most important family of transcription factors that transactivate lipogenic genes in order to increase FA and cholesterol syntheses. Under homeostatic conditions, SREBPs are synthesized in an inactive form and are attached to the ER membrane. Upon intracellular sterol shortage, SREBPs are proteolytically cleaved, whereupon they translocate to the nucleus and bind to sterol response elements (SREs). This binding of the activated SREBP to SRE DNA motifs governs the transcriptional control of key lipogenic metabolic enzymes, such as FASN and ACC (64–66).

### DNA Viruses

As discussed above, the first evidence of metabolic reprogramming upon HCMV infection was provided by Munger et al. Metabolic flux and MS analyses revealed that HCMV infection induces a glucose flux, which directly fuels FA synthesis (7, 16). In 2011, Munger's research group was able to show that HCMV infection facilitates the mTOR-dependent proteolytic cleavage of SREBP2 (67, 68). Other research groups provided additional evidence that SREBP1 cleavage is also required for optimal metabolic reprogramming toward lipogenesis to enable high-titer HCMV replication (69, 70). In those studies, the inhibition of SREBP proteolytic cleavage and of the downstream targets of SREBP-induced lipogenesis (e.g., ACC and FASN) impaired HCMV replication (16, 67, 69). Additionally, HCMV infection induces the expression of FA elongases (ELOVLs), which in turn leads to the accumulation of long-chain and very-long-chain FAs (VLCFAs) (68, 71) that are shuttled toward viral envelope production (71, 72). Among the family of FA elongases, ELOVL7 is increased more than 150-fold upon HCMV infection in an mTOR/SREBP-dependent manner (68). Remarkably, inhibition of ELOVL7 impairs HCMV replication, and this effect can be rescued upon ELOVL7 overexpression or VLCFA supplementation (68, 71). Additionally, HCMV-infected cells upregulate low-density lipoprotein receptor-related protein 1 (LRP1) in a SREBP-dependent manner, and interference with LRP1 disturbs the intracellular cholesterol availability (73). Besides this control of lipogenesis on a transcriptional level, HCMV can also directly increase ACC activity (67).

As mentioned above, KSHV infection induces a transformation of the host cell's glucose, glutamine, and fatty acid metabolism (27, 28). While infection-induced glycolysis and glutaminolysis prove essential for early steps of KSHV infection including genome replication, fatty acid synthesis appeared not to be involved in those processes. Instead, fatty acid synthesis is critical for virion assembly and the maturation of infectious particles, since KSHV-infected cells cultured in the presence of an ACC1 inhibitor produced only non-infectious intracellular virions (27). Other evidence provided by the same research group suggests that lipogenesis is required for KSHV survival and latent infection (28) (Figure 1).

### RNA Viruses

Likewise, perturbations of cellular lipid metabolism have proven vital for the *Flavivirus* replication cycle. Both DENV and West Nile virus (WNV) are known to be highly sensitive to the inhibition of ACC or FASN (74–78) as well as to interferences with cholesterol uptake (79), homeostasis (80), and biosynthesis (81, 82). An urgent need for *de novo* lipogenesis as well as changes in the intracellular lipid distribution and accumulation of unsaturated FAs have also been proposed to be essential prerequisites for DENV type 2 (DENV2) infection (75, 83). Gullberg et al. identified stearoyl-CoA desaturase 1 (SCD1, which catalyzes the rate-limiting step in the formation of unsaturated FAs) as a critical target that regulates the composition of intracellular membranes to induce a favorable microenvironment for optimal DENV2 replication and to sustain a high rate of infectious particle release (83). The pharmacologic inhibition of SCD1 interrupted the generation of monounsaturated FAs, such as oleic acid (C18:1) or palmitoleic acid (C16:1) (69, 83, 84), which consequently affected an optimal lipid membrane composition and membrane fluidity, leading to decreased viral replication efficiency in DENV2-infected cells. This detrimental effect of pharmacologic SCD1 inhibition on DENV2 replication has been expanded to several other *Flaviviridae* members, including four DENV serotypes, Yellow Fever Virus (YFV), Zika virus, and Japanese encephalitis virus (JEV) (83, 85). Therefore, the inhibition of ACC or FASN or that of more downstream lipid-modifying enzymes such as SCD1 may guide future therapies against *Flavivirus* infection (86, 87). Substantiating these findings, several groups have observed temporal changes in numerous lipid species, especially phospholipids, upon HCV infection (44), and a critical need for *de novo* ACC- and FASN-mediated FA synthesis to fuel viral replication (44, 88–90). More recently, metabolomic profiling revealed that unsaturated long-chain FAs, such as oleic acid (C18:1), specifically accumulated upon HCV infection, and that the accumulation of unsaturated FAs may influence the membrane composition and fluidity (84, 91, 92). Hofmann et al. demonstrated that the inhibition of FA elongases or desaturases restricted HCV replication (91). In their study, treatment with an inhibitor of Δ6-fatty acid desaturase (FADS2) impaired HCV virion production possibly through changes in the intracellular membrane composition, virion assembly, and morphogenesis (91, 92). Likewise, a liver-specific SCD1 inhibitor has been proposed for anti-HCV therapy following its proven efficacy in mice (84, 92–94). Similarly, inhibition of FASN with C75 reduced HCV replication *in vitro* (88). Other evidence suggests that currently used anti-HCV agents like ribavirin inhibit lipogenesis as a side effect, which may contribute to their antiviral properties (95, 96). Mechanistically, ribavirin suppresses the expression of lipogenic genes such as SREBP-1c, FASN, and SCD-1 in a retinoid X receptor α- and CCAAT/enhancer-binding protein α-dependent manner (95, 96). Statins, another class of drugs used in a wide number of patients due to their cholesterol- and lipid-lowering properties, exhibited an inhibitory effect on HCV replication probably due to the inhibition of the rate-limiting step of the mevalonate pathway, 3-hydroxy3-methyl-glutaryl coenzyme A reductase (HMG-CoA reductase) (97, 98).

Lipidomic analysis has also broadened our understanding regarding the metabolic reprogramming that ensues upon human coronavirus (HCoV) (99) and Middle East respiratory syndrome coronavirus (MERS-CoV) infections (99, 100). Yan at al. observed a striking rearrangement of the cellular lipid profile indicated by an accumulation of FAs (both saturated and unsaturated FAs) and phospholipids upon HCoV infection. The authors claimed that the *Coronaviridae* specifically fine-tuned the host lipid profile to achieve optimal viral replication (99). These findings were corroborated by a recent study that identified the pharmacologic targeting of SREBP (with the specific inhibitor AM580) as a promising means to inhibit MERS-CoV infection in multiple cell types *in vitro* and *in vivo* (100). Inhibition of the proteolytic processing of SREBP by AM580 caused the inhibition of several post-viral-entry steps, including reduced intracellular lipid droplet formation, reduced double membrane vesicle formation, and reduced palmitoylation of viral proteins (100), which potentially mirror the observations and conclusions made earlier by Yan et al. (99). Importantly, treatment with AM580 also restricted SREBP-dependent lipogenesis in influenza H1N1-infected cells, which resulted in the decreased palmitoylation of the surface glycoprotein hemagglutinin and ultimately impaired H1N1 replication (100).

Similarly, elevated levels of multiple long-chain mono- and polyunsaturated FAs have been associated with RV infection (39, 101). As discussed earlier, our group has recently shown that RV induces PI3K-dependent glucose uptake that feeds anabolic lipogenesis in primary human fibroblasts and HeLa cells (39). Another group recently confirmed our findings, using lipidomic technologies in primary human bronchial epithelial cells at different time points during a single replicative cycle of RV infection (i.e., ranging from 2–6 h post infection) (101). In accordance with our data, Ngyuen et al. observed an accumulation of FAs with long acyl chains in infected cells as compared with uninfected controls, as well as dynamic changes in the desaturation status of FA pools within the host cell. As a proof of concept, they treated the cells with several inhibitors of enzymes engaged in FA synthesis, elongation, and modification (including C75, an inhibitor of FASN), which resulted in a reduction in RV replication (101). Similarly, inhibition of FASN with a novel potent inhibitor (TVB-3166) decreased the replication of RV, respiratory syncytial virus (RSV), and human parainfluenza virus 3 (HPIV 3) (102). Confirming the essential need of FASN during viral replication, those observed effects could be rescued upon addition of exogenous palmitic acid (102). Altogether, both the upstream interference in the glucose flux (using 2-DG) (39) and the downstream inhibition of lipogenesis (101, 102) can serve as new therapeutic targets for treating RV- or RSV-induced respiratory infections.

During Chikungunya virus (CHIKV) infection, the FASN-mediated increase in the cellular lipid pool results in the increased palmitoylation of the virus's non-structural protein NsP1 at three cysteine residues by zinc finger DHHC domain-containing palmitoyltransferases (103). The palmitoylation of NsP1 is critical for CHIKV replication since it orchestrates capping of the virus's RNA (104–106). Therefore, the CHIKV induction of anabolic lipid synthesis via FASN in host cells generates an adequate substrate supply for the proper functioning of intracellular palmitoyltransferases. Confirming these observations, the inhibition of FASN was shown to impair CHIKV replication, which could be rescued upon exogenous palmitic acid supply (103).

Last, an up to 5-fold induction of FASN was also observed upon HIV-1 infection, translating into increased intracellular palmitic, oleic, and stearic acid pools (107). Although the authors were not able to delineate how those *de novo*-synthesized lipids fuel HIV-1 replication, they did show that FASN was exclusively required during the late stage of the viral replication cycle. This indicates a role for FASN in HIV-1-mediated viral budding or in post-translational modifications of HIV-1 structural proteins, such as the Gag protein (107). In line with this, existing evidence has proposed an essential role for several post-translational lipid modifications of HIV-1 structural proteins (108–111).

Hence, the life cycle of most viruses is closely linked to the composition of the cellular lipidome that defines the viral and cellular membrane composition, macromolecule synthesis, and post-transcriptional modification of viral proteins. In order to ensure sufficient substrate supply to enable the optimal replication of viral particles, viruses exploit host transcription factors and co-opt several enzymes engaged in *de novo* lipid synthesis and processing. The aforementioned lines of evidence suggest that lipid-based antiviral strategies may guide future antiviral therapies. In particular, the inhibition of SREBP cleavage and the targeting of FA-modifying enzymes (e.g., FA elongases and desaturases) represent promising targets for broad-spectrum antiviral metabolic intervention. However, the *in vivo* relevance of virus lipid interactions has yet to be determined, and further studies are urgently needed to better understand these processes.

## Conclusion and Outlook

We have elaborated on the various forms of virus interference with the host cellular metabolome. We have seen that although many of the induced changes follow similar patterns between different viruses, a distinct virus-specific fingerprint can nonetheless be found for each virus, which mirrors the needs of the respective pathogen for specific molecular compounds in the process of its replication.

Notwithstanding, most of our knowledge on the field consists of phenotypic characterizations of the impact of the infection on central pathways in host-cell metabolism, whereas our understanding of the mechanistic basis for these changes is far more limited. As we have seen, viruses have developed strategies as diverse as the activation of cytosolic signaling [e.g., PI3K (39) and CaMKK1/AMPK (9, 18) activation] or transcriptional regulation [e.g., activation of Myc (34, 35), ChREBP (21), SREBP (67–70, 100)]. The current data point toward a dichotomy between DNA and RNA viruses when looking at their respective strategies for host cell manipulation; that is, whereas the transcriptional control of key metabolic pathways was found for several DNA viruses (21, 34, 35), RNA viruses appeared to shape host-cell metabolism via post-transcriptional modifications (39), which are in line with the pace of the respective replication cycles.

As summarized in Table 1, our knowledge on the specific alterations induced by a given virus has resulted in numerous strategies to target viral replication with high efficacy in cell culture and *in vivo* models. However, because many of the established targets for metabolism-manipulating antivirals are central enzymes in cellular metabolism, future research will have to elaborate on whether the mentioned strategies can be translated into clinical applicability without causing major harm to unaffected host cells. Here, 2-DG in particular appears to be a promising compound, given its very well-established and favorable side-effect profile. Undoubtedly, further research in this dynamic area will help deepen our understanding of this interaction and might result in additional ways to impair viral replication by means of metabolic intervention. For instance, there are still major blind spots, particularly in our understanding of the mechanistic basis of RNA virus-induced alterations in cellular metabolism. Furthermore, there has been little research on the role of pattern recognition in the context of the above-mentioned adaptations. Additionally, many of the findings reported herein were generated in the context of highly specific cellular models, and differential modulations in different target cells (e.g., proliferating T cells) might result in adverse observations. We still have limited knowledge on the role of metabolism in the pathogenesis of a plethora of pathogenic viruses, which requires further research. Insights into these and other questions will help us to greatly advance our understanding of this crucial host–pathogen interaction and might sharpen our therapeutic arsenal to target this viral Achilles' heel.

**Table 1:** Strategies for metabolism-targeting interventions against different viruses.

**Virus**	**Compound**	**Target**	**Pathway**	**References**
HSV-1	2-DG	Phosphoglucose-isomerase	Glycolysis	(4, 9)
	STO-609	CaMKK	Ca^++^-sensing	(9)
	CB-839	Glutaminase	Glutamine metabolism	(35)
HCMV	STO-609	CaMKK	Glycolysis	(9, 19)
	Compound C	AMPK	Broad metabolic alterations	(18, 19)
	AICAR	AMPK	Broad metabolic alterations	(19, 112)
	2-DG	Phosphoglucose-isomerase	Glycolysis	(9)
KSHV	Oxamate	Lactat-dehydrogenase	Anaerobic glycolysis	(27)
	BPTES	Glutaminase	Glutamine metabolism	(27)
	TOFA	ACC1	Fatty acid metabolism	(27, 28)
VACV	BPTES	Glutaminase	Glutamine metabolism	(36)
	TOFA	ACC1	Fatty acid metabolism	(37)
	C75	FASN	Fatty acid metabolism	(37)
RV	2-DG	Phosphoglucose-isomerase	Glycolysis	(39)
	C75	FASN	Fatty acid metabolism	(101)
	TVB-3166	FASN	Fatty acid metabolism	(102)
RSV	TVB-3166	FASN	Fatty acid metabolism	(102)
HCV	MK8245	Stearoyl-CoA desaturase-1	Fatty acid metabolism	(93)
	SC-26196	Fatty acid Δ-6-desaturase	Fatty acid metabolism	(91)
	C75	FASN	Fatty acid metabolism	(88)
	CP640186	ACC	Fatty acid metabolism	(93)
	Ribavirin	SREBP-1c, FASN, stearoyl-CoA desaturase-1	Fatty acid metabolism	(95, 96)
	Statins	HMG-CoA reductase	Cholesterol synthesis	(97, 98)
DENV	C75	FASN	Fatty acid metabolism	(76)
	Cerulenin	FASN	Fatty acid metabolism	(76)
	MK8245	Stearoyl-CoA desaturase-1	Fatty acid metabolism	(85)
	A939572	Stearoyl-CoA desaturase-1	Fatty acid metabolism	(83)
HPIV 3	TVB-3166	FASN	Fatty acid metabolism	(102)
ZIKA	MK8245	Stearoyl-CoA desaturase-1	Fatty acid metabolism	(85)
WNV	C75	FASN	Fatty acid metabolism	(77)
	Cerulenin	FASN	Fatty acid metabolism	(77)
	TOFA	ACC	Fatty acid metabolism	(74)
JEV	MK8245	Stearoyl-CoA desaturase-1	Fatty acid metabolism	(85)
CHIKV	C75	FASN	Fatty acid metabolism	(103)
	Cerulenin	FASN	Fatty acid metabolism	(103)
YFV	A939572	Stearoyl-CoA desaturase-1	Fatty acid metabolism	(83)
Adenovirus	CB-839	Glutaminase	Glutamine metabolism	(35)
HIV	VU0359595	PLD-1	Glucose metabolism, nucleotide synthesis	(48)
	Fasnall	FASN	Fatty acid metabolism	(107)
MersCoV	AM580	SREBP	Fatty acid metabolism	(100)
Influenza A	AM580	SREBP	Fatty acid metabolism	(100)
	CB-839	Glutaminase	Glutamine metabolism	(35)

## Author Contributions

KM and GG wrote the article. JS and GZ carefully revised the manuscript and provided critical intellectual input. All authors agreed to the final version of the article.

### Conflict of Interest Statement

GG and JS are listed as inventors on a patent concerning 2-DG as an antiviral agent against rhinovirus infection. The remaining authors declare that the research was conducted in the absence of any commercial or financial relationships that could be construed as a potential conflict of interest.
